# Functional Connectivity Anomalies in Adolescents with Psychotic Symptoms

**DOI:** 10.1371/journal.pone.0169364

**Published:** 2017-01-26

**Authors:** Francesco Amico, Erik O’Hanlon, Dominik Kraft, Viola Oertel-Knöchel, Mary Clarke, Ian Kelleher, Niamh Higgins, Helen Coughlan, Daniel Creegan, Mark Heneghan, Emmet Power, Lucy Power, Jessica Ryan, Thomas Frodl, Mary Cannon

**Affiliations:** 1 Department of Psychiatry, Royal College of Surgeons in Ireland, Dublin, Ireland; 2 Trinity College Institute of Neuroscience, Trinity College Dublin, Dublin, Ireland; 3 Laboratory of Neuroimaging, Department of Psychiatry, Psychosomatic Medicine and Psychotherapy, Goethe University, Frankfurt, Germany; 4 Department of Psychology, Division of Population Health Sciences, Royal College of Surgeons in Ireland, Dublin, Ireland; Maastricht University, NETHERLANDS

## Abstract

**Background:**

Previous magnetic resonance imaging (MRI) research suggests that, prior to the onset of psychosis, high risk youths already exhibit brain abnormalities similar to those present in patients with schizophrenia.

**Objectives:**

The goal of the present study was to describe the functional organization of endogenous activation in young adolescents who report auditory verbal hallucinations (AVH) in view of the “distributed network” hypothesis of psychosis. We recruited 20 young people aged 13–16 years who reported AVHs and 20 healthy controls matched for age, gender and handedness from local schools.

**Methods:**

Each participant underwent a semi-structured clinical interview and a resting state (RS) neuroimaging protocol. We explored functional connectivity (FC) involving three different networks: 1) default mode network (DMN) 2) salience network (SN) and 3) central executive network (CEN). In line with previous findings on the role of the auditory cortex in AVHs as reported by young adolescents, we also investigated FC anomalies involving both the primary and secondary auditory cortices (A1 and A2, respectively).

Further, we explored between-group inter-hemispheric FC differences (laterality) for both A1 and A2. Compared to the healthy control group, the AVH group exhibited FC differences in all three networks investigated. Moreover, FC anomalies were found in a neural network including both A1 and A2. The laterality analysis revealed no between-group, inter-hemispheric differences.

**Conclusions:**

The present study suggests that young adolescents with subclinical psychotic symptoms exhibit functional connectivity anomalies directly and indirectly involving the DMN, SN, CEN and also a neural network including both primary and secondary auditory cortical regions.

## Introduction

Hallucinations and delusions, the classic symptoms of psychosis, are far more prevalent in the population than psychotic disorder [1]. Specifically, a meta-analysis of community-based studies found a median psychotic symptom prevalence of 17% in children aged 9 to 12 years and 7.5% in adolescents aged 13 to 18 years [2]. These symptoms are clinically important not only because they are associated with a relatively increased risk for psychotic disorder [3] but because they are strongly predictive of poor mental health outcomes more generally, including multi-morbid psychopathology [4–6], suicidality [7–9], neurocognitive impairment [10] and poor socio-occupational functioning [11, 12].

In recent years, considerable amount of research has been devoted to studying the pre-onset, or prodromal, phase of schizophrenia. This research includes the identification of putatively prodromal subjects using established criteria [13, 14] and the evaluation of ultra-high risk (UHR) for psychosis. Interestingly, magnetic resonance imaging (MRI) studies have shown that, prior to the onset of psychosis, UHR youths already exhibit brain abnormalities similar to those present in patients with schizophrenia [15–22]. In particular, resting-state (RS) functional connectivity (FC) MRI (rsfMRI) has shown anomalies in intrinsic neuronal activity generated by the brain of psychotic individuals [23] and specific brain activation patterns that distinguish normal visual imagery from auditory hallucinations [24]. However, little rsfMRI research has investigated the prodromal phase of psychosis and many questions still remain unanswered.

The default mode network (DMN) is a neural circuit that is thought to regulate internal thought monitoring [25–27], most commonly including the medial prefrontal cortex (MPFC), anterior and posterior cingulate cortices (ACC and PCC), inferior parietal cortex (IPC) and lateral temporal cortex (LTC) [23, 28]. A recent rsfMRI study on 39 adolescents aged from 12 to 20 years showed that activity in the DMN was unrelated to schizotypal trait expression, suggesting that the link between the DMN and schizotypy may be modified at later developmental stages of both FC and psychotic expression [5]. Another recent study on adolescents with 22q11 syndrome and psychotic symptoms, revealed that atypical connectivity in DMN, specifically within the left superior frontal gyrus region, correlated with prodromal symptom intensity and neuropsychological performances [29].

Recent research in a community sample of young people with psychotic symptoms suggests that decreased processing speed could be linked to aberrant functional connectivity within and between whole-brain neural systems, rather than indexing impairment in discrete neural networks [10, 30]. Direct evidence for this is gradually emerging. A study on a community sample of adolescents with psychotic symptoms by Jacobson McEwen and colleagues [31] suggests that a disruption in integration between distributed neural networks (particularly between prefrontal, cingulate and striatal brain regions) could be an important neurobiological feature of this population. In line with this view, recent rsfMRI research on young adults at risk for psychosis suggests that an aberrant coupling between the DMN and two other large-scale brain networks called “salience network” (SN)—anchored in the dorsal ACC (dACC) and right anterior insula (rAI)–and central executive network (CEN), a neural circuit that is activated during goal oriented activity that includes primarily the dorsolateral prefrontal cortex (DLPFC) and posterior parietal cortex (PPC [32] could be an important feature in the risk state [33]. This has been proposed as a “Triple Network Model” for the development of psychotic disorders [34–36]. The Triple Network Model gains support from several lines of evidence:

1. aberrant functional connectivity in the DMN correlates with the severity of hallucinations and delusions [37]. Within the DMN, abnormal FC between the MPFC and the PCC has been demonstrated even at the early stages of psychosis [38] and weaker connectivity between the ACC and the anterior component of the DMN has been shown to be associated with loss of insight [39]. Moreover, neuronal activity in the ACC is thought to mediate the expression of positive symptoms in schizophrenia [40] and FC anomalies involving the ACC are associated with reduced auditory discrimination in schizophrenic subjects [40]. Interestingly, Northoff and Qin [3] have proposed that elevated RS activity in the auditory cortex might be linked to an abnormal interplay between the auditory cortex and anterior cortical midline structures associated with the DMN. This has led to the hypothesis that in the brain of subjects with psychotic symptoms, there may be an abnormal interaction between the DMN, more in general, and the auditory cortex during RS [3], suggesting that abnormal functional connectivity between the auditory cortex and the DMN could play a role in the generation of auditory hallucinations [37], with within- and between-network connectivity anomalies developing gradually from childhood to adulthood.
2. The SN plays an important role in the generation of psychotic symptoms. In particular, it has been suggested that delusions and hallucinations may arise out of the aberrant attribution of salience to external and internal representations. For instance, delusions are implicated in the individual's effort to make sense of the aberrant salient experience and hallucinations reflect aberrant salience on the internal representations [41]. More recent rsfMRI research strengthens the role of the SN in schizophrenia suggesting that psychosis might arise from the failure of the insula to recruit prefrontal systems when salient or novel information becomes available [36].
3. Psychosis might emerge from an aberrant connectivity between the DMN, SN and CEN. In particular, rsfMRI data from schizophrenia patients indicate aberrant connectivity between the DMN and CEN as a denominator of auditory verbal hallucinations (AVHs) severity [35].
4. Perceptual inhibitory failure models of AVHs assume that speech is spontaneously generated in hallucinating individuals by an over-active primary auditory cortex and that this activity, possibly due to an altered connectivity in the auditory network [42], is able to propagate to higher levels of processing [3, 43]. In line with this model, a multicenter resting state study of individuals with AVHs reported less activation in the left primary auditory cortex during AVHs following the presentation of external tones [44], suggesting that the primary auditory cortex is “turned on” already during the resting state by showing increased endogenous activity. This suggests an orientation of perception towards internally-generated, rather than externally-generated (or stimulus-generated) activity.

Importantly, FC evidence also indicates cross-hemisphere dysconnectivity between primary and secondary auditory cortex in patients with schizophrenia [25], suggesting a disruption in multiple auditory functions, for example, the between-hemisphere integration of basic auditory (primary auditory cortex) and higher-order language processing abilities (secondary auditory cortex). In this regard, a recent fMRI study showed altered functional asymmetry in the primary auditory cortex and altered connectivity between temporal and limbic areas in the auditory network during RS in both patients with schizophrenia and high risk subjects (healthy subjects with family history of schizophrenia), which correlated with predisposition towards hallucinations, suggesting that reduced/altered hemispheric lateralization and reduced FC of the auditory network could be high risk trait markers of schizophrenia [42].

The aim of the present study was three-fold: 1) to explore putative FC anomalies involving the DMN, SN and CEN in drug-naive adolescents with psychotic symptoms; 2) to investigate the role of the auditory cortex (primary and secondary) in sub-clinical AVHs as reported by young adolescents; 3) to search for inter-hemispheric differences arising from aberrant connectivity of the auditory cortex (primary and secondary).

## Materials and Methods

### Participant Recruitment

A sample of 212 young people between 11 and 13 years old was recruited from primary schools in North Dublin and Kildare, Ireland, as part of the “Adolescent Brain Development Study” [1]. All 212 participants attended a diagnostic clinical interview with trained raters (I.K., S.R., M.H. and M.C.). For further details on the recruitment and interviewing, refer to Kelleher et al. [1]. Psychotic symptoms were assessed using the psychosis section of the “Schedule for Affective Disorders and Schizophrenia for School-Age Children” (K-SADS) [45]. This schedule is a well-validated, semi-structured research diagnostic interview for the assessment of current and lifetime DSM-IV Axis I psychiatric disorders in children and adolescents. The psychosis section contains questions designed to assess hallucinations and delusions. If any psychotic experience was reported, a full written account detailing the reported experience was taken. A consensus committee comprising an adult psychiatrist (M.C.), a child and adolescent psychiatrist (M.H.), and a psychologist (I.K.) met after the interviews to discuss the transcripts and determine whether any experiences elicited could be considered definite psychotic experiences or not. Factors associated with the experience, such as timing, content, frequency, attribution, severity, and distress, were taken into account in classifying experiences as definite or possible. The most common symptom reported was AVHs, which were present in more than 90% of those reporting symptoms [46]. A subsample of 100 participants with no contraindications to MRI agreed to take part in a subsequent study that took place 1 to 3 years after the original interview. Of the 100 individuals imaged, 28 adolescents 13 to 16 years old met criteria for the presence of a definite psychotic experience. These individuals were classified as the AVH group. From the remaining 72 participants, a group of 28 adolescents who had not reported psychotic experiences at the initial interview were chosen to match the AVH group for age (at the time of imaging), sex, and handedness. These individuals formed the healthy control group. After checking image realignment data, participants with motion parameters that exceeded 3 mm in any direction or 3.0° of any angular motion during the scan were excluded. For each exclusion (8 subjects/group), gender and age matching was adjusted. Altogether, this produced two groups of 20 age- and gender-matched participants/group (age range: 12–16 years), all right handed (Table 1). All control subjects were screened before imaging to exclude newly emerging psychotic symptoms. Ethical approval was obtained for the experimental protocol from the Medical Research Ethics Committee, Beaumont Hospital, Dublin and the School of Psychology, Trinity College, Dublin. Written parental consent and participant assent were obtained before the study.

**Table 1:** Demographic information per group with between-group significance (p-value) shown per measure as determined by student t-test.

Measure	AVH (N = 20)	Controls (N = 20)	*t-test (P* value)
Mean age (years)	14.2	14.1	0.81
(SD)	(1.28)	(1.29)	
Range	12–16	12–16	
Gender (M /F)	11/ 9	11 / 9	--
Handedness (R / L)	20/0	20/0	--

### Data Acquisition

Functional and structural images from each participant were obtained using a 3 T MRI scanner (Philips Achieva; PhilipsMedical Systems Netherland BV). The functional images were collected in single runs using gradient echo planar imaging (echo time, 28 ms; repetition time, 2000 ms; field of view, 131 mm; flip angle, 90°) sensitive to BOLD contrast (T2*weighting). A total of 39 contiguous sections with 3.2 mm thickness were acquired parallel to the anterior-posterior commissure plane (3 mm approximately isotropic resolution), providing complete brain coverage. The fMRI run included 180 volumes acquired continuously lasting 5.9 min in total. Of these, the first 5 volumes were excluded from analysis to minimize the effects of head movement. Structural data (for definitive atlas transformation) included a high-resolution sagittal, three-dimensional, T1-weighted, turbo gradient-echo sequence scan (echo time, 3.9 ms; repetition time, 8.5 ms; inversion time, 1060 ms; flip angle, 8°; 256 × 240 acquisition matrix, 1 × 1 × 1-mm voxels).

### Preprocessing of Functional Data

We used SPM software (SPM8, Wellcome Trust Centre for Neuroimaging [http://www.fil.ion.ucl.ac.uk/spm/software/spm8]) to preprocess fMRI data in the following steps: 1) compensation of systematic, section-dependent time shifts; 2) elimination of systematic odd- and even-section intensity differences due to interleaved acquisition; 3) rigid body correction for inter-frame head motion within and across runs. Data were excluded if motion parameters exceeded 3 mm in any direction or 3.0° of any angular motion during the scan, in line with previous studies [47–51]. Next, we coregistered the structural T1 image to the functional scans. Spatial normalization to standard 3 × 3 × 3 mm Montreal Neurological Institute (MNI) space was then applied to the functional images and to the structural image to allow for inter-subject analysis. Functional resting-state data were then spatially smoothed (full width at half maximum, FWHM) with an 8 mm kernel following standard procedures of SPM8.

### Functional Connectivity Analysis of Resting-State Activity

Using resting-state software [CONN; National Institutes of Health Blueprint for Neuroscience Research (http://www.nitrc.org/projects/conn)] to compute FC maps corresponding to a selected seed region of interest, we correlated the regional time course against all other voxels within the brain. CONN uses a CompCor strategy for physiological noise source reduction, first level General Linear Model for correlation and regression connectivity estimation, and second level random-effect analyses. Importantly, the CONN toolbox first implements an anatomical, component-based, noise correction strategy (CompCor) to identify and reduce physiological and other noise signals that are unlikely to be related to neural activity [52]. After regressing out Compcor-identified noise, the resulting BOLD time series were band-pass filtered (0.008–0.09 Hz) to further reduce noise and increase sensitivity.

To address the spurious correlations in resting-state networks caused by head motion, we identified problematic time points during the scan using the “Artifact Detection Tools” in CONN (ART, www.nitrc.org/projects/artifact_detect/) a toolbox that allows detection of outliers in global signal intensity as well as translation and rotational movement parameters. Specifically, an image was defined as an outlier (artifact) image if the head displacement in x, y, or z direction was greater than 1.5 mm from the previous frame, or if the rotational displacement was greater than 0.034 radians from the previous frame, or if the global mean intensity in the image was greater than 3 standard deviations from the mean image intensity for the entire resting scan. The output matrices of SPM movement and threshold outliers generated ART were then entered into CONN as first-level covariates.

Based on previous findings [5–7, 37, 38, 53], we explored connectivity involving three different networks: 1) the DMN, 2) SN and 3) CEN. In line with our goal to explore the role of the auditory cortex in AVHs as reported by young adolescents, we also investigated FC anomalies involving the primary and secondary auditory cortices (A1 and A2, respectively). Correlation maps were produced by extracting the BOLD time course from a seed region of interest with a 5 mm radius and then by computing the correlation coefficient between that time course and the time course from all other brain voxels. Coordinates for seed regions of interest were automatically identified by CONN, whereas coordinates of the centre of voxel clusters showing significant FC with the chosen seed region were identified (region name and its coordinates) using the Automated Anatomical Labeling (AAL) toolbox in SPM8 [54]. Seed regions of interest were extracted: 1) from the DMN, the ACC (Brodmann area 33), was chosen for its possible role in the manifestation of sub-clinical positive symptoms in high risk subjects [8, 55] and after considering previous evidence suggesting that FC anomalies involving the ACC are associated with reduced auditory discrimination in schizophrenic subjects [8]; 2) on the basis of previous FC research [34, 36], the dorsal ACC (dACC, Brodmann area 32) and the insula (Brodman area 13) were chosen as seed regions for the SN; 3) as suggested by a similar study in adult patients with schizophrenia [36], the DLPFC (Brodmann areas 9 and 46) was chosen as seed region for the CEN; 4) in line with previous research supporting a role of the auditory cortex in the generation of AVHs, [3, 25, 43, 44] which could as well result from its abnormal FC with the DMN [3, 43], we also chose A1 (Brodmann areas 41 and 42) and A2 (Brodmann area 22) as seed regions for our analysis; 5) finally, in line with previous research [42] inter-hemishperic FC differences (i.e. laterality) involving the auditory cortex (A1 and A2) were explored. To do so, a between-source (i.e., left vs right A1 or left vs right A1) 2 x 2 mixed ANOVA group by laterality interaction analysis was run in CONN (second level analysis) and positive/negative functional correlations with seed areas explored in SPM8.

### Statistical Analysis of MRI Data

Analysis of fMRI data was run in CONN and SPM8 (http://www.fil.ion.ucl.ac.uk/spm) using 2-sample t-tests to determine significant differences in FC between AVH participants and controls using both uncorr.ected and family-wise error (FWE) whole-brain-corrected threshold of P < .05. Since we analyzed connectivity of three different networks, the threshold was reduced from p < .05 to p < .016. Condition (patients vs. controls) was used as covariate for second level analysis. Both “between-conditions” and between-source effects were investigated in CONN with a multivariate/repeated-measures analysis. Specifically, the second-level model is a general linear model that includes as regressors the selected terms in the “subject effects” list. The outcome variable is the within-subjects linear combination(s) of effects specified by the “between-conditions” and”between-sources” contrasts, applied to the first-level connectivity-measure volumes (for the seed-to-voxel analyses) or to the first-level connectivity-measure matrix (for the ROI-to-ROI analyses).

Several sources of spurious variance along with their temporal derivatives then were removed from the data by linear regression, such as signal from regions centered in the white matter, cerebrospinal fluid, and movement. CONN implements the component-based noise-correction method strategy for physiological and other noise-source reduction [56]. This method has the advantage of not requiring external monitoring of physiological fluctuations. Compared with methods that rely on global signal regression, the component-based noise reduction method allows for interpretation of anticorrelations because there is no regression of the global signal within this method. This approach may enhance the sensitivity and specificity of positive correlations and produce comparable negative correlations [11]. This regression procedure removes fluctuations unlikely to be involved in specific regional correlations.

## Results

### Demographics (Table 1)

There was no difference between participants scanned and not scanned in age [χ2(3, *N* = 211) = 0.51, *p* = .91)], sex [(χ2(1, *N* = 210) = 1.25, *p* = .26)], handedness [(χ2 (1, *N* = 157) = 0.18, *p* = .66)], presence of psychotic symptoms [(χ2 (1, *N* = 211) = 0.83, *p* = .36)], socioeconomic status [(χ2 (1, *N* = 165) = 1.76, *p* = .18)], personal psychiatric history [χ2 (1, *N* = 208) = 0.003, *p* = .95)] or family psychiatric history [(χ2 (1, *N* = 206) = 1.55, *p* = .21)]. Also, between the subjects that were included in the study (n = 20/group), there was no group difference in head motion. More specifically, we found no group difference in maximum head displacement [beta = -0.14 T(38) = -0.91, p = 0.81 (two-sided p = 0.37)] and average head motion [beta = 0.0015 T(38) = 0.08, p = 0.46 (two-sided p = 0.93)].

### Seed-To-Voxel Functional Connectivity (Table 2)

**Table 2:** Functional connectivity (FC) comparison between subjects with auditory verbal hallucinations (AVH) and controls in the default mode network (DMN), central executive network (CEN) and primary auditory cortex (A1)/secondary auditory cortex (A2). ACC: anterior cingulate cortex; dACC: dorsal ACC; CS: calcarine sulcus; DLPFC: dorsolateral prefrontal cortex; GR: gyrus rectus; IC: insular cortex; IFO: inferior frontal operculum; IOC: inferior orbitofrontal cortex; ITC: inferior temporal cortex; LG: lingual gyrus; MOC: middle occipital cortex; medial superior frontal cortex; OC: olfactory cortex; PCG: precentral gyrus.

Network	Seed	KE	P(clust)	P(peak)	Area	FC	X	Y	Z
DMN	left ACC	2766	-	0.001*.	left ITC	<	-46	+32	+36
	right ACC	5219	0.01 FWE	0.001*.	right SMFC	<	+4	+38	+48
		4833	0.01 uncorr.	0.001 **	right putamen	>	+34	-4	0
CEN	right DLPFC	4128	0.001 FWE	0.001*	left IFO	<	-42	+8	+22
SN	right dACC	5003	0.001 FWE	0.001*	left PCG	<	-26	-14	+72
Auditory	left A1(BA 41)	6905	0.001 FWE	0.001*	left GR	>	-6	+30	-26
	right A1(BA 41)	7638	0.001 FWE	0.001*	right Crus I	<	+52	-66	-26
	left A1 (BA 42)	5659	0.001 FWE	0.001*	right OC	<	+16	+12	-16
		73719	0.01 FWE	0.001*	left LG	<	-14	-90	0
	right A1(BA 42)	11673	0.01 FWE	0.001*	left MOC	<	-42	-86	0
	left A2	4405	0.001 FWE	0.01*	right IOC	<	+36	+34	-2
		11673	0.001 FWE	0.001*	CS	<	-10	-90	-2
		6517	0.001 FWE	0.001*	left SFC	>	-24	+46	+40
	right A2	5163	0.01 FWE	0.001*	left LG	<			

*uncorrected

**FWE

Default Mode Network (DMN): The AVH group had weaker connectivity between the left ACC and the left inferior temporal cortex [*KE* = 2766; p (peak-level, uncorr.) < 0.001; T = 4.05; Z = 3.7; x = -48, y = -38, z = -22]. Also the AVH had weaker connectivity between the right ACC and the right superior medial frontal cortex [*KE* = 5219; p (FWE, cluster-level) < 0.01; p (peak-level, uncorr.) < 0.001; T = 3.9; Z = 3.6 x = 4, y = 38; z = 48]. Finally patients had greater connectivity between the right ACC and the right putamen [*KE* = 4833; p (FWE, cluster-level) < 0.01; p (peak-level, uncorr.) < 0.001; T = 4.07; Z = 3.7; x = 34, y = -4, z = 0].

Central Executive Network (CEN): Patients had weaker connectivity between the right DLPFC and the left inferior frontal operculum [*KE* = 4128; p (FWE, cluster-level) < 0.01; p (peak-level, uncorr.) < 0.001; T = 3.8; Z = 3.5; x = -42, y = 8, z = 22].

Salience Network (SN): In the AVH group, there was weaker positive FC between the right dACC and the left precentral gyrus [*KE* = 5003; p (FWE, cluster-level) < 0.001; T = 4.6; Z = 4.1; p (peak-level, uncorr.) < 0.001; x = -26, y = -14, z = 72]. No between-group difference was found when the right or left insula was considered as seed region.

Auditory Cortex (A1 and A2): When compared with the control group, the AVH group had greater connectivity between the left A1 (BA 41) and the left gyrus rectus [*KE* = 6905; p (FWE, cluster-level) < 0.001; T = 4.2; Z = 3.8; Z = 3.7; p (peak-level, uncorr.) < 0.001; x = -6, y = 30, z = -26). Weaker connectivity was found in the AVH group between the right A1 (BA 41) and the right Crus I (*KE* = 7638; p (FWE, cluster-level) < 0.001; T = 3.8; Z = 3.5; p (peak-level, uncorr.) < 0.001; x = 52, y = -66, z = -26). In the AVH there was also weaker connectivity between the left A1 (BA 42) and the right olfactory cortex [*KE* = 5659; p (FWE, cluster-level) < 0.001; T = 4.2; Z = 3.8; p (peak-level, uncorr.) < 0.001; x = 16, y = 12, z = -16] or the left lingual gyrus (*KE* = 73719; p (FWE, cluster-level) < 0.01; T = 3.5; Z = 3.3; p (peak-level, uncorr.) < 0.001; x = -14, y = -90, z = 0). In the AVH group there was also weaker connectivity between the right A1 (BA 42) and the left middle occipital cortex (*KE* = 11673; p (FWE cluster-level) < 0.01; T = 4.3; Z = 3.9; p (peak-level, uncorr.) < 0.001; x = -42, y = -86, z = 0). The AVH group had also reduced connectivity between the left A2 and the right inferior orbitofrontal cortex [*KE* = 4405; (FWE, cluster-level) < 0.001; T = 4.3; Z = 3.9; p (peak-level, uncorr.) < 0.01; x = 36, y = 34, z = -2] or the left calcarine sulcus [(*KE* = 8189; p (FWE, cluster level) < 0.001; T = 3.8; Z = 3.5; p (peak-level, uncorr.) < 0.001; x = -10, y = -90, z = -2). The left A2 also had greater connectivity with the left superior frontal cortex in the AVH group when compared to controls [*KE* = 6517; p (FWE, cluster-level) < 0.001; T = 5.2; Z = 4.5; p (peak-level, uncorr.) < 0.001; x = -24, y = 46, z = 40. In the AVH group there was also reduced connectivity between the right A2 and the left lingual gyrus [*KE* = 5163; p (FWE, cluster-level) < 0.01; T = 3.7; Z = 3.4; p (peak-level, uncorr.) < 0.001; x = -18, y = -42, z = -8].

### Laterality

A between-source comparison of A1 activity across all subjects showed positive FC between the left A1 (BA 41) and left Rolandic operculum [p (FWE, cluster-level) < 0.001, KE = 16274; p (FWE, peak-level) < 0.001, T = 31.31, Z = >8; x = -48, y = -28, z = 14] as well as between the right A1 (BA 41) and right A2 [p (FWE, cluster-level) < 0.001, KE = 4170; p (FWE, peak-level) < 0.001, T = 22.91, Z = > 8; x = 52, y = -28, z = 8]. Positive connectivity was also found between the left A1 (BA 42) and left A2 [p (FWE, cluster-level) < 0.001, KE = 4735; p (FWE, peak-level) < 0.001, T = 18.20, Z = > 8; x = -64, y = -24, z = 12]. Further, activation of the right A1 (BA 42) was positively associated with the activation of the right A2 [p (FWE, cluster-level) < 0.001, KE = 2086; p (FWE, peak-level) < 0.001, T = 20.38, Z = >8; x = 68, y =—24, z = 8].

In the control group, the left A1 (BA 41) was positively connected with the left Rolandic operculum [p (FWE, cluster-level) < 0.001, KE = 6818; p (FWE, peak-level) < 0.001, T = 24.33, Z > 8; x = -44, y = -26, z = 14] and the right A1 (BA 41) with the right A2 [p (FWE, cluster-level) < 0.001, KE = 13505; p (FWE, peak-level) < 0.001, T = 14.91, Z = 6.88; x = 52, y = -28, z = 12]. There was a positive functional correlation between activity in the left A1 (BA 42) and activity in the left A2 [p (FWE, cluster-level) < 0.001, KE = 8334; p (FWE, peak-level) < 0.001, T = 14.34, Z = 6.78; x = -64; y = -26, z = 16]. Moreover, the right A1 (BA 42) was positively coupled with the right A2 [p (FWE, cluster-level) < 0.001, KE = 6056; p (FWE, peak-level) < 0.001, T = 13.52, Z = 6.63; x = 66; y = -32, z = 12].

In the AVH group, there was greater positive functional connectivity between left A1 (BA 41) and the left A2 [p (FWE, cluster-level) < 0.001, KE = 2919; p (FWE, peak-level) < 0.001, T = 24.7, Z > 8; x = -50, y = -28, z = 12], whereas activity in the right A1 positively correlated with activity in the right A2 [p (FWE, cluster-level) < 0.001, KE = 9251; p (FWE, peak-level) < 0.001, T = 19.4, Z = 7.5, x = 52, y = -28, z = 8]. More positive coupling was found between the left A1 (BA 42) and the left A2 [p (FWE, cluster-level) < 0.001, KE = 16954; p (FWE, peak-level) < 0.001, T = 14, Z = 6.73, x = -64, y = -24, z = 12] as well as the right A1 (BA 42) and the right A2 [p (FWE, cluster-level) < 0.001, KE = 27565; p (FWE, peak-level) < 0.001, T = 21.1, Z = 7.7, x = 68, y = -24, z = 10].

A group x laterality interaction analysis (i.e. contrasts: controls > AVH or AVH > controls) revealed no between-source (left A1 vs. right A1) FC difference between the AVH group and the control group.

Overall, activation of the right A2 was positively coupled to the right middle temporal gyrus [p (FWE, cluster-level) < 0.001, KE = 38991; p (FWE, peak-level) < 0.001, T = 13, Z > 8; x = 70, y = -46, z = 8] and the right medial superior frontal gyrus [p (FWE, cluster-level) < 0.001, KE = 14583; p (FWE, peak-level) < 0.01, T = 6.4, Z = 5.2; x = 12, y = 44, z = 56].

In the control or the patient group, there was no difference between the activation of the left and right A2. A between-group comparison (i.e. contrasts: controls > AVH or AVH > controls) revealed no between-source (left A2 vs. right A2) FC difference.

## Discussion

The present study revealed a number of important FC differences between young adolescents with subclinical psychotic symptoms and control subjects matched for age, gender and handedness, strengthening the role of multiple network dysconnectivity in psychosis [6, 57]. The first important result involved the DMN. Specifically, in the AVH group, we detected weaker connectivity between the ACC and the inferior temporal cortex a region where volumetric reductions have been found in patients with chronic schizophrenia [58] and, most importantly, whose connectivity with the DMN has been shown to be altered in UHR subjects [59]. The ACC also had weaker connectivity with the superior medial frontal cortex, in line with previous research indicating that anomalies in frontal cortical areas are associated with both high risk for schizophrenia [28] and psychotic symptoms [23]. Further, this result confirms other RS data indicating an implication of the medial frontal cortex in aberrant DMN activation, with effects on abstract thinking and negative symptoms [60]. The results are also interesting in view of the connectivity hypothesis [27, 34], which proposes a role for the SN in schizophrenia, an intrinsic large-scale network including the ACC and frontal cortical areas that could be involved in the coordination of the DMN and task-related circuits.

We also found altered functional connectivity between the ACC and the putamen, in line with previous MRI studies showing structural [61] and functional [62] basal ganglia abnormalities in patients with schizophrenia and that dysfunction of the putamen may be linked to delusional symptoms [63]. These results might also support our recent findings indicating that white matter microstructural insults in fronto striatal tracts can be found in young adolescents reporting psychotic symptoms [22]. Importantly, the greater connectivity we detected between the ACC and the putamen might provide further support to the aberrant salience hypothesis of schizophrenia [62], suggesting that altered cortico-striatal-thalamic neurocircuitry is responsible for chaotic firing of dopaminergic neurons in the striatum, leading to aberrant assignment of salience to innocuous stimuli.

In support of previous research [7], we found abnormal FC between the dACC, a region of the SN that is thought to play a role in internal stimulus salience detection, and the precentral gyrus, a region that is part of a neural system involved in the motor expression of language [64] as well as in auditory monitoring and feedback of speech [65]. Previous research has shown FC anomalies involving the precentral gyrus in schizophrenia [66, 67], although whether these anomalies are directly linked to AVH still needs to be determined.

Interestingly, a recent fMRI study by Diederen et al. [68] comparing psychotic subjects and non-psychotic subjects with AVHs revealed a common activation pattern involving the precentral gyrus and the insula, suggesting a link between precentral gyrus and SN activation during the generation of AVH in non-psychotic subjects. The present study might support this hypothesis also suggesting an involvement of the dACC.

Another interesting finding was the reduced connectivity between a region of the CEN (DLPFC) and the operculum, a brain structure that is adjacent and intimately connected [69] to the insula (SN). This result could be in line with the hypothesis linking the development of psychotic disorders to anomalies in the synchronization between DMN, SN and CEN [34–36]. However, no connectivity changes were found between the CEN and DMN of the AVH group, suggesting that these might reflect a more pronounced expression of the psychosis phenotype. In this context, a recent fMRI study on 20 medication-free adolescents with brief psychotic episodes has shown that disengagement of the DMN was concomitant to the experiencing of hallucinations [70], suggesting that aberrant FC anomalies between the DMN and the CEN are more likely to be detected during a psychotic episode rather than during a symptom-free period. Hence, given that our participants with subthreshold psychotic experiences were not specifically or intentionally scanned during a psychotic experience or episode, putative FC anomalies between the DMN and CEN in the AVH group cannot be completely ruled out.

A unique finding of this study was the altered connectivity between the auditory cortex and frontal (A1: gyrus rectus; A2: inferior orbitofrontal cortex, superior frontal cortex) as well as prefrontal (A2: inferior orbitofrontal cortex) cortical regions in the AVH group, suggesting both direct and indirect relationships with the DMN. Altered FC between the DMN (i.e. ACC) and the gyrus rectus has been demonstrated in subjects with early onset of schizophrenia [71] and reduced blood volume in basal orbitofrontal cortical areas was detected in high risk individuals [72]. Further, previous research has shown a link between increased vulnerability to developing AVHs and altered FC between the primary auditory cortex and orbitofrontal cortex. The present study extends this finding, demonstrating abnormal connectivity between secondary auditory and orbitofrontal cortical areas in high risk subjects [72]. Indirectly-related to DMN activity was also the weaker connectivity detected between the A1 and Crus I, whose coupling with DMN areas has previously been found to be altered in UHR subjects [73] and in drug-naive patients with schizophrenia at rest [74].

A FC anomaly was also found between the A1 and the olfactory cortex, suggesting an involvement of the DMN and SN through the close anatomical relationship of the olfactory region with orbitofrontal and insular areas, respectively. In psychotic disorders, including schizophrenia, olfactory deficits can be detected at the very early stage of the disease, but progress very little, if any [75]. Speculatively, given the role of olfaction in attributing an emotional valence to the environment [76, 77], an aberrant connectivity between olfactory and primary auditory cortical regions could result in altered emotional salience of the auditory stimuli processed by the brain. Finally, the analysis of FC with the A1 and A2 regions revealed between-group differences with the occipital cortex. More specifically, connectivity anomalies were found between the A1 region and the middle occipital cortex/lingual gyrus as well as between for the A2 region and the calcarine/lingual gyrus. Changes in the occipital lobe have been previously demonstrated in schizophrenia [78, 79] and in individuals with genetic vulnerability to developing schizophrenia [80]. Further, abnormal connections between frontal and occipital areas have been demonstrated in support of the CEN-SN dysfunctional interaction hypothesis in schizophrenia [81]. The profile we found for the A1 and A2 regions, might suggest that altered FC between auditory, olfactory and occipital cortical regions could underpin the disruption of multimodal stimulus processing. However, whether this dysfunction was related to the generation of AVHs in the high risk subjects recruited for the present study still needs to be determined.

Altogether, our results are in line with previous data showing a link between altered connectivity in the auditory cortex/network and (auditory) psychotic symptoms in individuals with schizophrenia [25, 42] as well as with a recent RS study on young adolescents with psychotic symptoms indicating an implication of frontal areas [31]. However, while there is evidence that, in adults, these symptoms are influenced by aberrant integration in language processing [39, 82], our study shows also a direct involvement of primary auditory cortical regions. Findings from research employing fMRI as well as electroencephalography (EEG) are consistent with the idea that activity in auditory processing areas increases during AVHs when compared to silent rest. Of particular interest, is the research showing that the primary auditory cortex is mainly involved [83], potentially explaining the realistic nature of the hallucinatory experience. Moreover, these results strengthen the role of frontal regions in AVHs [40, 84–86] and support previous research suggesting that AVHs could arise from elevated resting state activity in the auditory cortex, which might be related to abnormal modulation of the auditory cortex by anterior cortical midline structures associated with the DMN [3]. The connectivity anomalies involving both the auditory cortex and the dACC are also interesting in the framework of the “directed effort network-representational network” desynchonization hypothesis of psychosis [70], according to which failure of the directed effort network (including the dorsal and posterior/anterior cingulate cortices, the auditory cortex, and the hippocampus) to synchronize with the representational network (a neural circuit including the frontal pole, temporal pole, and fronto-insular cortex) could play a role in the generation of psychotic symptoms [53]. Under this perspective, our findings might suggest that connectivity between the directed effort network and the representational system could be compromised in the much broader extended psychosis phenotype, incorporating members of the population with psychotic symptoms as well as the narrower schizophrenia population.

Finally, the results from the laterality analysis are very interesting. In both the AVH and the healthy control group normal FC between A1 and A2 [87] was preserved. Similarly, in both groups, the A2 was found to be functionally coupled with the middle temporal cortex, a region reported to be vulnerable to structural changes in patients with schizophrenia [88]. Further, in both in the AVH group and controls, activation in the (left) A1 correlated with the activation of the (left) Rolandic operculum, a cortical area whose abnormal activity has been previously shown in prodromal patients [89] and in patients with first-episode psychosis [90].

## Conclusions

Young adolescents with subclinical psychotic symptoms exhibit FC anomalies directly and indirectly involving the DMN, SN, CEN. Further, altered FC between auditory, olfactory and occipital cortical regions could underpin the aberrant multimodal processing and salience attribution of auditory stimuli. Whether the dysconnectivity detected can play a role in the generation of AVHs and/or psychotic episodes should be investigated in future studies. The strengths of our study are the use of treatment-naive, non-help-seeking adolescents from a community setting. However, in view of the small group sizes, findings would require replication in a larger sample.
